# Gastric bezoars secondary to mixed infection with *Sarcina ventriculi* and G + bacilli: a case report

**DOI:** 10.1186/s12879-024-09560-1

**Published:** 2024-07-12

**Authors:** Tao Wang, Dan Xiang

**Affiliations:** 1 Department of Pathology, Ya’an People’s Hospital, Ya’an, Sichuan China; 2 Department of Clinical Laboratory, Ya’an People’s Hospital, Ya’an, Sichuan China

**Keywords:** *Sarcina ventriculi*, Gastric bezoar, G + bacilli

## Abstract

*Sarcina ventriculi* is a bacterium with a specific histological morphology and infection can present with symptoms such as abdominal pain, nausea, vomiting and occasionally fatal complications. Delayed gastric emptying is regarded as the most significant risk factor for infection. Its pathogenicity is currently unknown and treatment options are inconsistent. Here we report a case of gastric bezoars secondary to a mixed infection of *Sarcina ventriculi* and G + bacilli, which is diagnosed by a pathological biopsy.

## Introduction

*Sarcina ventriculi* is widespread in nature and is commonly found in plants, water and soil [1], but human infections with *Sarcina ventriculi* are rare and mixed infections are even rarer [2]. It is one of the rare bacteria that can be identified on pathological biopsy due to its peculiar tetrameric or octameric morphology [2]. We report a case of a diabetic patient with gastric bezoars secondary to a gastric infection with *Sarcina ventriculi* and gram-positive(G+) bacilli, which resolved after an antibiotic-free regimen.

## Case report

**Fig. 1:**
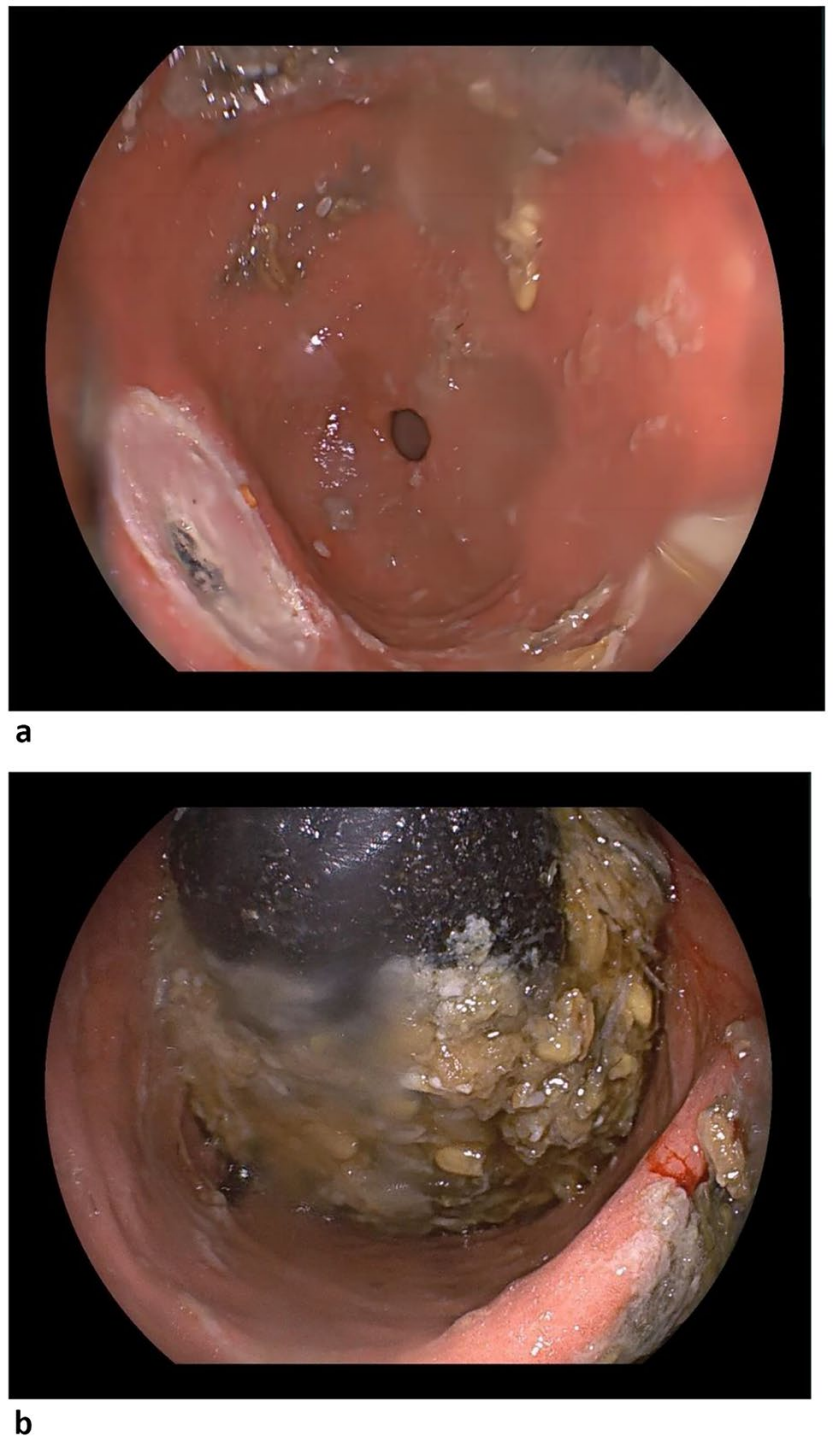
Initial gastroduodenoscopy. Ulcers in the gastric sinus (**a**) and food retention in the gastric corpus(**b**)

44 year Chinese man with poorly controlled diabetes presented with abdominal pain and melena for more than two weeks. The patient’s vital signs appeared to be stable. The physical examination revealed deep tenderness in the upper abdomen. Fasting blood glucose was measured at 11.4 mmol/L and glycaemic haemoglobin at 9.00%. In addition, the haemoglobin level was measured to be 9.3 g/dL. White blood cell count and liver enzymes were normal. Initial gastroduodenoscopy showed multiple ulcers in the gastric sinus, along with significant gastric residue[Figure 1a, b]. The C13 breath test was positive.

**Fig. 2:**
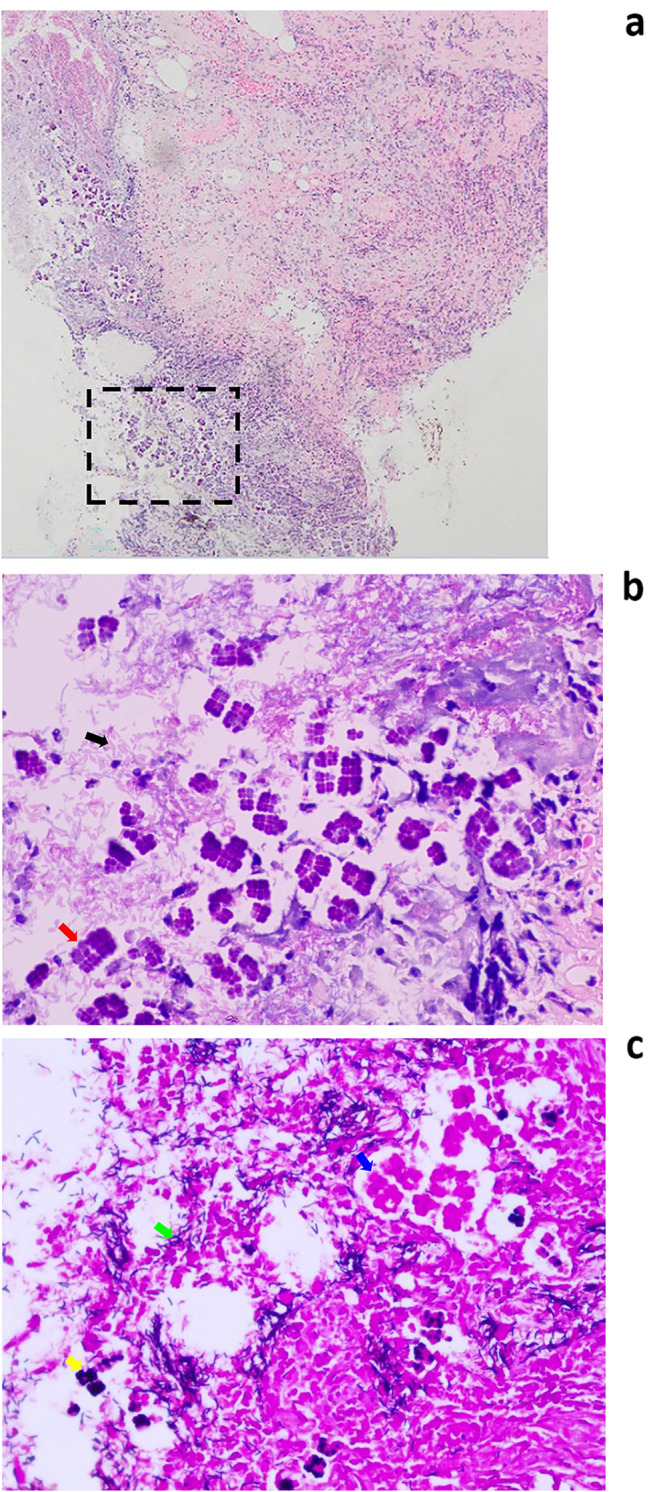
(**a**) Low magnification histological section of gastric ulcer tissue, H&E stain×100. (**b**) The magnified area within the dashed box in Fig. 2a, *Sarcina ventriculi* cells(red arrow) surrounded by bacilli(black arrow), H&E stain×600. (**c**) Gram stain×400, *Sarcina ventriculi* were partly positive (yellow arrow) and partly negative (blue arrow), and bacilli were positive (green arrow)

**Fig. 3:**
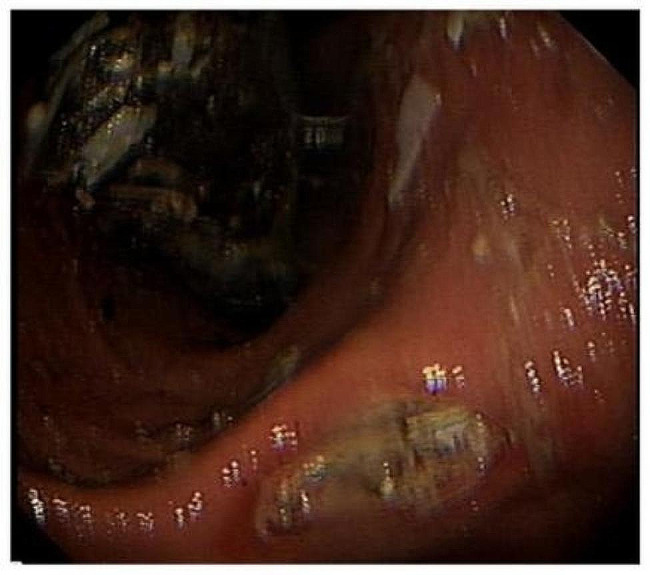
Second gastroduodenoscopy showed food bezoar and ulcers in the gastric sinuses

The biopsy taken from the ulcer site was stained with hematoxylin eosin and Gram stain, revealing active inflammation of the mucosa with necrosis. Two distinct types of bacteria were identified on the mucosal surface. The first type of bacterium is cocci, with a diameter of 1.5-3 microns. They exhibit a refractory nature and basophilic staining with hematoxylin-eosin. These cells aggregate into characteristic groups of four, eight, or higher-order polymers, forming encapsulated or cuboidal shapes. Upon contact, the cell walls flatten, anchoring together to create tetrad or octad structures, which collectively produce the distinctive “dynamite bundle” appearance. The results of Gram staining are variable, with some cells exhibiting a positive reaction and others negative. The second type of bacterium is a G + bacillus with a length of approximately 5–6 μm, exhibiting no other specific morphology. Both bacteria exhibit non-invasive growth(Fig. 2a, b,c). No further bacterial culture and species identification was performed. The final combined diagnosis was gastric ulcer with mixed *Sarcina ventriculi* and G + bacilli infection. The patient was initially treated with omeprazole and empagliflozin for hyperglycaemia. However, after more than 50 days of treatment without symptom relief, a second gastroduodenoscopy was performed. This revealed the presence of several large bezoars in the gastric corpus and fundus, along with additional ulcers in the gastric sinus[Figure 3]. The largest bezoar measured approximately 5 cm or more in diameter and could not be fully visualized. The bezoars were subsequently removed during endoscopy. Subsequently, the patient was treated with a combination of omeprazole and rebapatide, along with arginine-zinc recombinant insulin for glycaemic control. After 12 months of follow-up, the patient’s symptoms had significantly improved. Repeat gastroduodenoscopy and gastric biopsy showed good gastric motility, healed gastric ulcers, and the absence of *Sarcina ventriculi*.

## Discussion

*Sarcina ventriculi* is a microorganism with a unique morphology that lacks spores and motility. It can grow and reproduce in acidic environments, with cell morphology changing and becoming distorted at pH greater than 3. At pH greater than 8, it can produce spores [3]. It relies on the fermentation of carbohydrates to produce energy, resulting in the production of carbon dioxide and ethanol [3]. In 1842, John Goodsir made the initial discovery of *Sarcina ventriculi* in human vomit [4]. Subsequent studies revealed its predilection for the stomach. Based on its morphological characteristics and the sample source, it was classified as *Sarcina ventriculi* in the Sporosarcina genus. The microorganism was previously misidentified as *Clostridium ventriculi*, but has since been renamed *Sarcina ventriculi* [5]. Although it was once thought to be Gram-positive, recent studies suggest that its Gram staining is poor, and it may be more appropriate to call it Gram-variable [6]. Diagnosis of *Sarcina ventriculi* often relies on recognition of its distinctive morphology on pathological examination. Polymerase Chain Reaction and sequencing of 16 S rRNA, as well as pyruvate decarboxylase genes, can enhance diagnostic accuracy. However, these molecular confirmations are generally not considered necessary [2].

*Sarcina ventriculi* is often reported as causing human infections in the stomach, and in some cases, it coexists with Helicobacter pylori infection [7]. It has also been detected in blood, urine, esophagus, duodenum, lungs, and occasionally in asymptomatic individuals. Gastric infections are mainly associated with gastritis, gastric ulcers, indigestion, emphysematous gastritis, and gastric perforation [8]. Studies have shown that delayed gastric emptying, caused by conditions such as gastroparesis, gastric reconstruction surgery, pyloric stenosis, immunodeficiency, and poorly controlled diabetes, is associated with the colonisation and growth of *Sarcina ventriculi* in the gastrointestinal tract [9–11]. Delayed gastric emptying can cause food retention, which can extend the reproduction time and provide ample nutrition. The gases produced by *Sarcina ventriculi* metabolism can further exacerbate delayed gastric emptying [2]. If indigestible food is ingested in this situation, it may form bezoars in the stomach, which can further induce or aggravate gastric mucosal damage and even cause gastrointestinal obstruction. Reports of gastric bezoars following *Sarcina ventriculi* infection are rare, suggesting that the occurrence of gastric bezoars is not an inevitable consequence of *Sarcina ventriculi* infection. In this case, gastric bezoars were detected more than 50 days after the diagnosis of gastritis, gastric ulcers, gastric retention, and mixed infection of *Sarcina ventriculi* and G + bacilli. Further research is required to establish whether *Sarcina ventriculi* contributes to the development of gastric bezoars.

Currently, there is no established treatment protocol for *Sarcina ventriculi* infection. Treatment should be based on the patient’s accompanying diseases. Fasting or antibiotic treatment should not be considered mandatory. Animal laboratory studies have shown that isolated *Sarcina ventriculi* is sensitive to (fluoro)quinolones, macrolides, penicillins, and tetracyclines [12]. The reported results suggest that Sarcina ventriculi infection can be resolved through glycaemic control, treatment with Proton pump inhibitors and pro-gastric motivational drugs, without the need for antibiotic therapy. This phenomenon has also been confirmed in previous studies [13, 14]. However, due to the potential fatal risks, such as gastric perforation and emphysematous gastritis, some cases list antibiotic treatment as a preferred option [15]. Whether to use antibiotic treatment should be considered comprehensively based on the patient’s condition.

The increasing number of reports suggests that *Sarcina ventriculi* affects gastric health and is not merely a bystander. However, further research is needed on why it causes serious complications in some individuals but not in others.

## Conclusion

*Sarcina ventriculi* is a rare pathogen that affects the stomach’s health, with delayed gastric emptying being the most significant risk factor. The initial diagnosis of *Sarcina ventriculi* infection relies on microscopic observation of pathological biopsy. Treatment options should be determined based on a combination of concomitant diseases. Recognising the potential serious complications of *Sarcina ventriculi* infection and avoiding misdiagnoses will aid patient recovery.

## Data Availability

No datasets were generated or analysed during the current study.
